# QSAR Models for Predicting the Antioxidant Potential of Chemical Substances

**DOI:** 10.3390/jox15030080

**Published:** 2025-05-25

**Authors:** Sofia Ghironi, Edoardo Luca Viganò, Gianluca Selvestrel, Emilio Benfenati

**Affiliations:** 1 Laboratory of Environmental Toxicology and Chemistry, Department of Environmental Health Sciences, Istituto di Ricerche Farmacologiche Mario Negri IRCCS, 20156 Milan, Italy; edoardo.vigano@marionegri.it (E.L.V.); emilio.benfenati@marionegri.it (E.B.); 2 Environmental Sustainability for Industrial and Health Systems Unit, Department of Environmental Health Sciences, Istituto di Ricerche Farmacologiche Mario Negri IRCCS, 20156 Milan, Italy; gianluca.selvestrel@marionegri.it

**Keywords:** antioxidants, QSAR, DPPH, food, food supplements, machine learning, in silico

## Abstract

Antioxidants are widely studied compounds with significant applications in the nutraceutical and dietary industries. To enable the rapid screening of large libraries of substances for antioxidant activity and to provide a useful tool for the initial evaluation of substances of interest with unknown activity, we developed Quantitative Structure–Activity Relationship (QSAR) models to predict the antioxidant potential of chemical substances. We started from a dataset of 1911 antioxidant substances, retrieved from the AODB database by selecting the DPPH (1,1-diphenyl-2-picrylhydrazyl) radical scavenging activity assay and the experimental value of the half-maximal inhibitory concentration. Different machine learning algorithms were applied to build regression models, and the goodness-of-fit of each model was assessed using the statistical parameters of R squared (R^2^), the Root-Mean-Squared Error, and the Mean Absolute Error. The Extra Trees model outperformed the other models in both internal and external validations, achieving the highest R^2^ of 0.77 and the lowest RMSE on the test set. Gradient Boosting and eXtreme Gradient Boosting also achieved promising results with R^2^ values of 0.76 and 0.75, respectively. Given these results, we developed an integrated method that not only outperformed the individual models, achieving an R^2^ of 0.78 on the external test set, but also provided valuable insights into the range of predicted values.

## 1. Introduction

Antioxidants are well-studied natural or synthetic substances able to delay, prevent, or inhibit the oxidation of other substances or substrates, with applications in different fields, including nutrition and food supplements. This class of active compounds is known for its primary function: the ability to counteract oxidative stress, which is the product of an increase in Reactive Oxygen Species (ROS). ROS, such as superoxide (O_2_^−^) and hydroxyl radicals (OH⋅), are physiologically produced by organisms as intermediates during metabolic reactions and are highly reactive molecules due to the presence of unpaired electrons. To reduce ROS, the human body employs endogenous defenses that can be categorized into enzymatic antioxidants, including Superoxide Dismutase and Catalase, and non-enzymatic molecules, such as reduced Glutathione [1,2]. Although small amounts of ROS are physiologically required in cellular signaling and immune responses, their concentration determines their hazard; an excess can damage key biomolecules such as lipids, proteins, and DNA, contributing to the development of numerous chronic diseases such as cancer, cardiovascular diseases, and neurodegenerative disorders, as well as accelerating the aging process. External factors could interfere with the endogenous antioxidant systems; for instance, an unhealthy diet, smoking, drinking alcohol, and environmental pollution could increase the formation of ROS [3,4,5,6]. Balancing antioxidants and ROS is crucial considering the relationships between oxidative stress, cellular damage, various diseases, and aging. Therefore, the intake of antioxidants through the diet or concentrated formulations such as food supplements and nutraceuticals may be determinative in handling oxidative stress. For this reason, specific health claims govern their promotion in food products to inform consumers of the benefits of antioxidant intake through a specific food [7,8]. Given their wide range of applications, natural and synthetic antioxidants are also commonly found within foods as additives. For instance, butylated hydroxyanisole (BHA) and butylated hydroxytoluene (BHT) are synthetic antioxidants that are widely used in the food industry as preservatives to inhibit lipid oxidation and extend shelf life. Among the wide range of natural and synthetic compounds with reported antioxidant activity, particular interest has emerged around those derived from agricultural and industrial by-products (e.g., grape pomace, olive leaves), especially in the context of sustainability and circular economy. This approach transforms waste materials into resources for nutraceutical, cosmetic, and food applications. Thus, these bioactive compounds have been investigated for their potential health-promoting properties, along with the possibility of reducing waste and generating high-value products [9,10]. A wide variety of analytical techniques are currently available to assess antioxidant activity, including in vitro, biochemical, in vivo, and ex vivo models. Others, such as those evaluating DNA damage protection, can provide additional insights into antioxidant effects even when not specifically designed for that purpose. These techniques offer valuable insights but can be time-consuming and resource-intensive. In silico approaches are increasingly being adopted as predictive tools to overcome these limitations. Indeed, in this work, we develop a battery of in silico models for prediction of the antioxidant potential of small molecules. In silico methods are part of the new approach methodologies (NAMs) and offer an alternative to the more traditional in vivo approach. They include techniques like Read-Across, Physiologically Based Pharmacokinetic (PBPK) modeling, and Quantitative Structure–Activity Relationship (QSAR) modeling. In particular, QSAR models work by correlating the properties of a molecule to its biological interactions, providing an efficient way to predict the activity of substances under investigation [11,12]. Situations involving the assessment of large numbers of compounds could certainly benefit from the in silico approach, saving on costs, time, and the use of laboratory animals [13]. Furthermore, these methods provide greater environmental protection than the more widespread in vivo and in vitro experimentation.

In silico models have previously been developed to predict antioxidant activity, focusing on various assays and endpoints [14,15]. In these works, antioxidant activity was treated as a binary classification endpoint, due to the wide variety of assays considered. However, for our purposes, we chose to maintain very specific conditions in the experimental data, reducing noise from different assays and sources of information, and considered this endpoint as a regression, which we consider more valuable for our purposes than a binary classification. We developed QSAR regression models to predict the antioxidant potential of small molecules by estimating their half-maximal inhibitory concentration (IC50) values.

## 2. Materials and Methods

### 2.1. Collection and Processing of the Data

Following an extensive literature review, data were retrieved from the AODB database [16,17], a comprehensive collection of 98,313 small molecules, peptides, and proteins tested for antioxidant activity. The AODB database for small molecules was filtered based on specific parameters of interest, including (a) the assay type, (b) the class of substances (excluding peptides), and (c) the experimental value provided as a quantitative figure. Among the different assays accessible within the database, the 1,1-diphenyl-2-picrylhydrazyl (DPPH) radical scavenging activity assay was selected to build our models. The DPPH radical scavenging activity assay [18] is a colorimetric test introduced by Blois in 1958 to evaluate antioxidant activity. Experimentally, the substance to be tested is added to the violet-colored radical DPPH solution (Equation (1)). If the tested substance exhibits antioxidant properties, it quenches the DPPH radical, resulting in a color change from violet to yellow and a consequent decrease in absorbance.

(1) $$DPPH\cdot +AH\to {DPPH}_{2}+A$$

Determining the absorbance of the solution provides a measure of the antioxidant power of the substance under investigation.

This assay was chosen for its widespread use and the extensive amount of available data. A large volume of data is crucial for training high-quality machine learning models as it enables better generalization and improves model performance. The IC50 value was chosen as the preferred experimental value because of its easy interpretation, as it indicates the concentration required for a compound to inhibit 50% of the DPPH radical activity. A lower IC50 value suggests a higher antioxidant capacity, as a lower amount of the substance is needed to neutralize free radicals. To ensure consistency, only experimental IC50 values were taken into account, excluding undefined records (e.g., those marked with “<” or ”>” or given as ratios). The keyword “DPPH” was used to gather the data of interest; given the large number of entries associated with this assay, amounting to 3790, we focused only on substances obtained through an experimental procedure based on a 30-min time frame [19]. This selection also ensured more uniform and consistent data, better suited for modeling.

For the 146 entries with missing temporal information, a manual check was performed, screening the related publications; in this way, we could fill in most of the incomplete entries, obtaining a dataset of 2832 compounds. Each of them was identified by the International Chemical Identifier (InChI), which is a standard and structure-based chemical identifier [20]; the Simplified Molecular Input Line Entry System (SMILES) [21], a notation system that allows the chemical structure of a substance to be represented in alphanumeric form; the molecular weight; the DOI of the paired publication; the assay description; the experimental value; and the ChEMBL ID [22]. Each compound was associated with its experimental IC50 value for antioxidant activity, which was then standardized to molar (M) units. After this extensive data preprocessing step, the dataset was curated following generally used techniques. In particular, salts were neutralized, counterions and inorganic elements were excluded, stereochemistry was removed, and the SMILES data were canonized. Chemicals with a molecular weight higher than 1000 Da were removed, according to the definition of small molecules [23]. In this way, we eliminated all peptides. Duplicates were removed using both the InChIs and the canonical SMILES data. Initially, the InChIs were evaluated, the duplicates were grouped, and the mean (μ) and standard deviation (σ) of the associated experimental values were calculated. Then, the coefficient of variation (CV) (Equation (2)), given by the ratio of the standard deviation to the mean, was computed as well. In this way, a cut-off of 0.1 was chosen to remove duplicates with a higher CV; the remaining duplicates were instead retained by considering the mean of the experimental value. The same procedure was repeated on the processed SMILES data, specifically SMILES data without stereochemistry and which had undergone canonization.

(2) $$CV=\frac{\sigma }{\mu }$$

The experimental data were also transformed into the negative logarithmic form (pIC50) with the goal of achieving a more Gaussian-like distribution, which can help improve modeling performance. The statistical parameter of skewness was calculated to assess changes in data distribution. Following these steps, 1911 compounds were collected from the AODB database and used to develop a battery of regression models.

### 2.2. Molecular Descriptors

Molecular descriptors (MDs) provide a quantitative means of mathematically representing various molecular properties. They are among the most widely used methods for encoding chemical information in QSAR studies. These descriptors can be categorized into classes, such as constitutional, geometrical, and physicochemical, based on the properties they encode [24]. MDs enable us to define each compound using thousands of numerical indices representing different chemical properties, such as polarizability, steric hindrance, or molecule shape. In other words, MDs are quantitative representations of chemicals, capturing various aspects of their chemical structure, properties, or behavior. The Mordred Python package V1.2.0 [25] was selected to calculate the molecular descriptors for our dataset. Mordred is a descriptor calculation software application that can calculate more than 1800 2- and 3-dimensional MDs, including topological descriptors. The calculated descriptors were then preprocessed through standardization using the StandardScaler from the scikit-learn library in Python. This widely used method scales descriptors by subtracting the mean value and dividing by the standard deviation, ensuring that each descriptor contributes equally to the model and improving the stability and performance of machine learning algorithms.

#### Feature Selection

Feature selection is a critical step in developing a machine learning model, as it helps identify the best subset of variables to represent the data. By reducing the number of variables and retaining only the most informative features, both the calculation time and data noise can be significantly reduced, thereby improving the model’s performance.

For feature selection, two univariate feature selection methods were used and combined. Univariate feature selection works by selecting the best features based on univariate statistical tests. It can be seen as a preprocessing step to an estimator. The first method computed the F-value between each feature and the target, identifying the most relevant descriptors based on the top k scores.

The second method was mutual information (MI) analysis. This technique measures the mutual dependence between two random variables and employs nonparametric methods based on entropy estimation using k-nearest neighbor (k-NN) distances. Features ranked with high scores by both methods were selected for modeling. The feature selection was performed using the scikit-learn 1.4.0 Python package.

### 2.3. Model Development and Validation

In our work, the data were split randomly, with 80% used for training and 20% reserved as a holdout set to test the models on unseen data. The models were developed using Python 3.11, scikit-learn 1.4.0, RDKit 2023.9.4, pandas 2.2.0, mordred 1.2.0, and imblearn 0.0.

Different regression algorithms were taken into account: Extra Trees, Gradient Boosting, XGB (eXtreme Gradient Boosting), Random Forest, k-NN, Adaptive Boosting (Adaboost), Decision Tree, Ridge, Elastic Net, Bayesian Ridge, and Lasso. Wherever needed, the hyperparameters of the algorithms were tuned to select the best hyperparameters for the model. This procedure aimed to improve the model performance by finding the optimal configuration setting for the algorithm. It was performed by applying an exhaustive grid search over a predefined set of hyperparameter values. In this way, we evaluated all possible combinations and selected the one that gave the best performance based on a specified metric, the correlation coefficient (R^2^) (Equation (3)), which is a measure of a model’s goodness-of-fit. The correlation coefficient ranges between 0 and 1 and is used to assess how well the independent variables explain the variation in the dependent variable.

The models with the best hyperparameters were then passed through a series of steps to validate the results. Internal and external validation were the methods used to assess the models’ generalizability and reliability, which play a crucial role in determining how well a model performs on new, unseen data.

In our work, internal validation was conducted using a 10-fold cross-validation. This approach involves splitting the training set into 10 equal subsets, or “folds”. The model is then trained and validated 10 times, with each iteration using a different subset as the validation set, while the remaining nine subsets form the training set. The model is retrained in each fold and evaluated on the held-out subset. This process provides a robust estimate of the model’s performance across various parts of the dataset.

The mean of the R^2^ values from each fold in the cross-validation was calculated, and a confidence interval was determined using Student’s t-distribution with an alpha value of 0.05. This provided an overall measure of the model’s accuracy and generalizability, helping to ensure that the model was not overfitted to any specific subset of the data. This method allowed us to assess the model’s stability and reliability within the internal training dataset, providing a sound basis for its performance before proceeding to external validation.

The external validation instead consisted of predicting a holdout set with data never seen by the model [26]. For the internal and external validations, the goodness-of-fit of every model was evaluated by calculating the statistical parameters of R^2^ (Equation (3)); the Root-Mean-Squared Error (RMSE) (Equation (4)), used to assess the difference between the predicted values and the experimental ones; and the Mean Absolute Error (MAE) (Equation (5)), which is the absolute difference between the predicted values and the experimental ones [27]. These metrics are defined by the following equations:

(3) $$R^{2}=1-\frac{\sum_{i=1}^{n}{\left(y_{i}-{\hat{y}}_{i}\right)}^{2}}{\sum_{i=1}^{n}{\left(y_{i}-\bar{y}\right)}^{2}}$$

(4) $$RMSE=\sqrt{\frac{\sum_{i=1}^{n}{\left(y_{i}-{\hat{y}}_{i}\right)}^{2}}{n}}$$

(5) $$MAE=\frac{\sum_{i=1}^{n}\left|y_{i}-\hat{y_{i}}\right|}{n}$$

where $y_{i}$ is the experimental value, ${\hat{y}}_{i}$ is the predicted value, $\bar{y}$ is the mean of the dependent value, and n is the number of data points.

#### Applicability Domain

According to the third OECD principle [28], a QSAR model should be associated with a defined applicability domain (AD). The AD of a QSAR model defines its boundaries within its structural and response space. This validation criterion restricts the model’s use to accurate predictions for test samples that have structural similarities to the training substances on which it was built.

Different methods were adopted to define the AD of the model, particularly the bounding box method and a distance-based method. Through the bounding box method, it is possible to determine the AD as a multi-dimensional box consisting of the data from the training set, with each side of the box corresponding to the range given by the minimum and the maximum value of each descriptor. Conversely, the distance method allows us to define the AD by evaluating the distance of the data in the test set from the centroid of the training set data.

## 3. Results and Discussion

**Figure 1:**
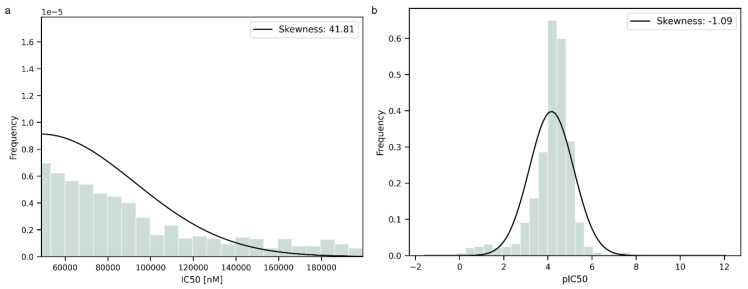
(**a**). Data distribution before logarithmic transformation. (**b**). Data distribution after logarithmic transformation.

Prior to the modeling step, the data were transformed into negative logarithmic form to normalize their distribution. To further evaluate the distribution of the data, the statistical parameter of skewness was computed. The data were improved by the transformation, as can be seen from the changes in skewness in Figure 1a,b. The original data exhibited a significantly higher skewness value than the transformed data, indicating an asymmetric distribution. The transformed data had a negative value of skewness near 0, suggesting that the distribution was more symmetric and closer to a normal distribution than the original one but with a longer tail on the left side.

**Figure 2:**
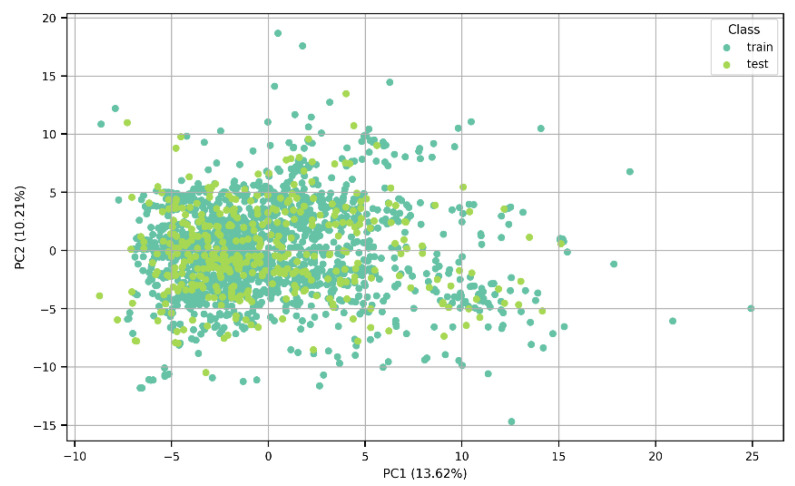
A PCA plot showing the distribution of the data between the training and test sets.

The algorithms were trained on a training test consisting of 1528 molecules and tested on a test set of 383 molecules. The split into training and test sets can be appreciated in the Principal Component Analysis results shown in Figure 2, in which a lack of clear clustering can also be noted. Additionally, the percentage variance was calculated; PC1 explained 13.62% of the total variance of the data, while PC2 captured 10.21% of the variability. The two components accounted for 23.83% of the variation in the dataset.

**Figure 3:**
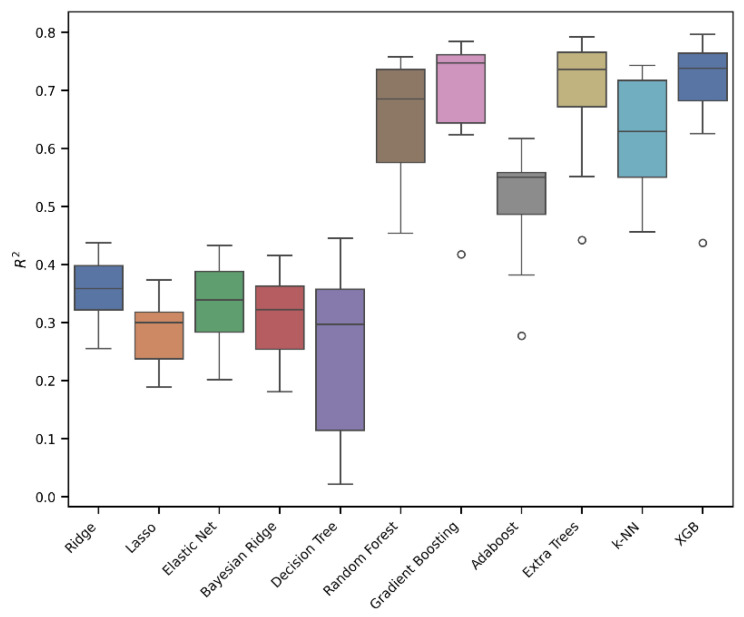
An evaluation of the regression models’ performance in 10-fold cross-validation. The box plot presents the interquartile ranges (Q1–Q3).

Eleven models were developed using the Extra Trees, Gradient Boosting, XGB (eXtreme Gradient Boosting), Random Forest, k-NN, Adaboost, Decision Tree, Ridge, Elastic Net, Bayesian Ridge, and Lasso algorithms. In Table 1, the results of 10-fold cross-validation are reported for each of the eleven models, while the box plots for the most relevant models are shown in Figure 3.

As can be seen from Figure 3 and Table 1, the best-performing models were Extra Trees, Gradient Boosting, and XGB. In particular, the Extra Trees Regressor outperformed the other models, achieving the highest R^2^ of 0.77, along with the lowest RMSE of 0.45 and MAE of 0.31. Conversely, the Lasso model showed the weakest performance, with an R^2^ value of 0.31. Gradient Boosting and XGB also achieved promising performances with R^2^ values of 0.76 and 0.75, RMSEs of 0.46 and 0.47, and MAEs of 0.32 and 0.33. Based on the results obtained, it can be concluded that a linear model is not the most effective approach for describing and predicting our data; instead, an ensemble tree approach, like Extra Trees, Gradient Boosting, or XGB, is preferable.

**Figure 4:**
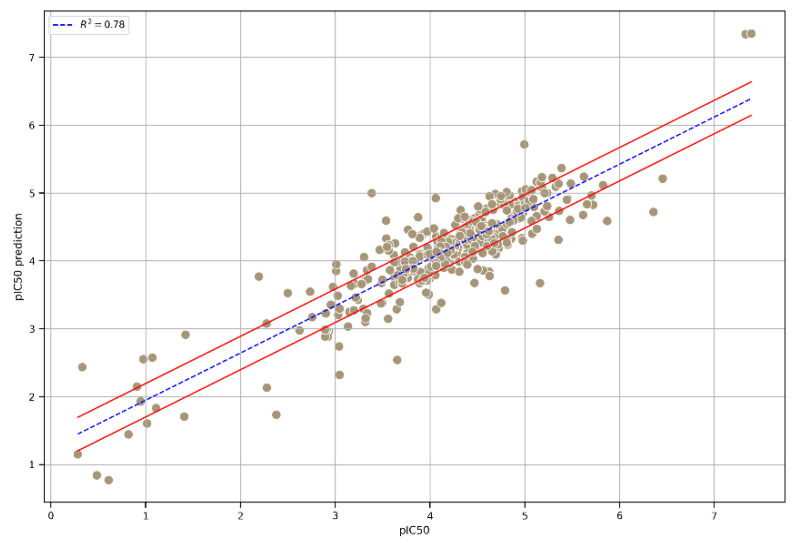
A plot of the correlation between the experimental pIC50 values and the pIC50 values predicted using the integrated model. The interval, indicated by the red lines, was calculated as the mean of three times the standard deviation of the predictions from each model used in the integrated model.

To further enhance predictability and robustness, we chose an integrated model that combined the three top-performing models. As displayed in the correlation plot (Figure 4), the performance of the three models, namely, Extra Trees, GB, and XGB, in terms of R^2^ was similar, ensuring greater prediction consistency. Moreover, the statistical parameters R^2^, RMSE, and MAE reached satisfactory values on the integrated model’s external test set (Table 2), achieving an R^2^ value of 0.78.

In the literature, prior studies have presented in silico models for this property, but they simply used classification methods [14,15]. Thus, we cannot compare our results with those previously published. We notice that predicting a continuous value, as in our case, is much more demanding than a binary classification, which may more easily accommodate errors and variations within the same class. A continuous value is much more informative than an active/not active representation, because it gives a clear indication about the potency of the effect.

The applicability domains were determined using two techniques: centroid-based and bounding box methods. To more rigorously evaluate the model’s predictive performance, compounds outside the AD were excluded from the analysis, minimizing the influence of unreliable predictions. As a result, particularly for the centroid-based method, we observed a slight increase in R^2^ values, suggesting a stronger correlation between the predicted and experimental data (Table 3). The exclusion of substances outside the AD—14 for the bounding box and 26 for the centroid-based method—reduced the number of substances that could be evaluated.

### 3.1. Case Study

To show the potential application of the developed models, we selected Urolithin A as a case study. This compound was selected because it was not present in either the training or test set, ensuring an unbiased assessment of the models’ predictivity. Urolithin A is a metabolite produced through the bacterial fermentation of the naturally occurring polyphenol Ellagic Acid in the intestinal lumen, and it is well known for its beneficial properties [29,30]. Using the SMILES representation of Urolithin A as its input, our consensus model predicted an IC50 value of 31 ± 6 mg/L. To validate this prediction, we compared it with experimental data from the literature [31], which indicated a measured IC50 of 35.5 µg/mL determined using the DPPH assay. Our predicted IC50 value was similar to the one reported by Marchese et al. [31], highlighting our models’ potential in estimating the antioxidant activity of small molecules.

### 3.2. Feature Importance

**Figure 5:**
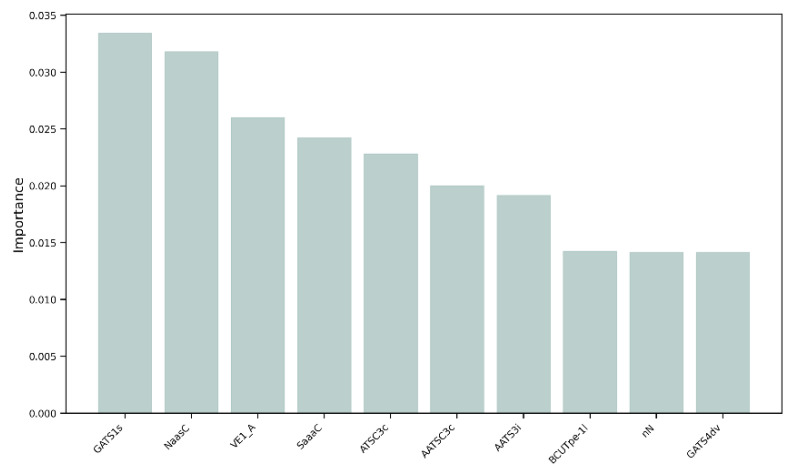
Feature importance of the 10 most influential molecular descriptors in the development of the models.

**Figure 6:**
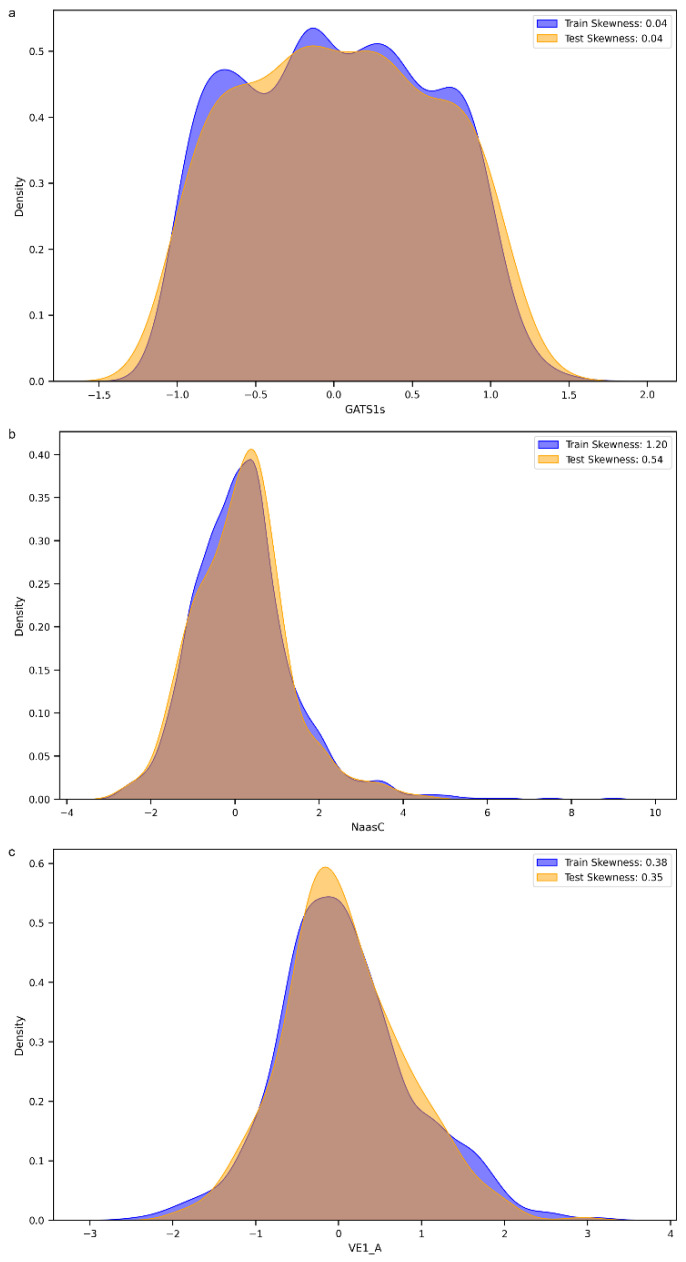
(**a**). Distribution of the GATS1s molecular descriptor across the training and test sets. (**b**). Distribution of the Naasc molecular descriptor across the training and test sets. (**c**). Distribution of the VE_1A molecular descriptor across the training and test sets.

We decided to perform a more thorough analysis by evaluating the most important features, i.e., the variables that mattered most in the development of the models, within the 152 selected MDs. As shown in Figure 5, the variable with the greatest weight was the 2D descriptor “GATS1s”, namely, Geary autocorrelation of lag 1 weighted by I-state [32]. “NaasC” or “Number of atoms of type aasc”, which is an electrotopological state index [33], was the second most important feature. The third most important MD was “VE1_A”, which stands for the coefficient sum of the last eigenvector (absolute values) from the adjacency matrix [32]. These three descriptors pertain to the electronic charge distribution and molecular geometry, reflecting critical aspects of the mechanisms by which molecules exert their antioxidant activity, such as electron transfer and affinity for radical regions. As previously mentioned, antioxidant activity primarily occurs through the ability of a molecule to donate or accept electrons, with the goal of stabilizing a radical species. When the distribution of values of these three most important MDs within our dataset was evaluated, a skewness of 0.04 was observed for GATS1s on both the training and test sets (Figure 6a), indicating an almost symmetric distribution. The two distributions showed good overlap, and different peaks in the training set highlighted the possible presence of clusters within the data. A different distribution was obtained for NaasC, with a more positive skewness (Figure 6b); in both the training and test sets, a single peak was visible. It is also worth pointing out the difference in skewness between the two curves: for the curve calculated on the training set, there was a skewness of 1.20, while for the curve calculated on the test set, it was 0.54. Regarding VE1_A, a slightly positive distribution was obtained in both the training and test sets, with skewness of 0.38 and 0.35, respectively, and a pronounced peak (Figure 6c).

### 3.3. Applications and Limitations

The developed QSAR models are valuable in silico tools for identifying chemical substances with potential antioxidant activity. By facilitating the rapid screening of compound libraries, these models can be applied in the early stages of Research and Development (R&D) processes within the nutraceutical and food industries. Their application can decrease the number of candidate compounds needing further experimental testing, thus reducing costs and accelerating the R&D pipeline. Additionally, the models can be used to better characterize novel compounds when limited experimental data are available. Although the models show strong predictive performance, they also present certain limitations, several of them originating from the assay used for their development. While the DPPH assay is widely employed to evaluate the antioxidant activity of chemical substances, it has well-known weaknesses. For example, it is highly sensitive to experimental conditions such as light exposure, temperature, and reaction time. Furthermore, the assay does not account for biological factors like the bioavailability or metabolism of the tested chemical, limiting its relevance for in vivo applications. Besides assay-related limitations, QSAR models are also subject to inherent constraints regarding their applicability domain. Indeed, our model, like all in silico models, is strictly related to the set of substances used to build the model.

## 4. Conclusions

Antioxidants have a major role in protecting DNA, lipids, and proteins from the damage caused by ROS and are recognized as important substances due to their significant applications in the nutraceutical and dietary fields. Thus, we decided to develop in silico models to predict the antioxidant activity of small molecules and assist in identifying potential substances that could be applied in the health support context. In the present study, a battery of eleven QSAR models was developed to predict the antioxidant potential of small molecules through their IC50 values. Three out of the eleven regression models—namely, Extra Trees, GB, and XGB—demonstrated good predictive performance, particularly Extra Trees. Given these results, we built an integrated model combining Extra Trees, GB, and XGB that outperformed the individual models and provided valuable insights into the range of predicted values. Antioxidant compounds are becoming increasingly important in the dietary supplement, food, and cosmetics industries, and the proposed approach provides reliable in silico models for evaluating antioxidant activity to explore even large sets of substances. The developed models represent a significant improvement in assessing the antioxidant potential of small molecules, offering the advantage of using regression instead of binary classification. Additionally, by reducing the need for laboratory testing, these models facilitate evaluations of antioxidant activity while offering ethical benefits and savings in terms of both cost and time.

## Figures and Tables

**Table 1:** Results from the regression models for an 80-20 data split, reporting the MAE, RMSE, and R^2^ for both the external test set and cross-validation. The mean results of 10-fold cross-validation are reported, with the 95% confidence interval calculated using Student’s t-distribution.

Algorithm	MAE	RMSE	R^2^	R^2^ in 10-Fold Cross Validation Median Value	R^2^ in 10-Fold Cross Validation
Extra Trees	0.31	0.45	0.77	0.74	0.72 ± 0.06
Gradient Boosting	0.32	0.46	0.76	0.75	0.72 ± 0.05
XGB	0.33	0.47	0.75	0.74	0.73 ± 0.04
Random Forest	0.33	0.48	0.74	0.69	0.65 ± 0.08
k-NN	0.33	0.50	0.72	0.63	0.63 ± 0.07
Adaboost	0.47	0.61	0.58	0.55	0.53 ± 0.08
Decision Tree	0.46	0.67	0.49	0.30	0.26 ± 0.10
Ridge	0.51	0.70	0.44	0.36	0.35 ± 0.04
Elastic Net	0.53	0.73	0.40	0.34	0.33 ± 0.06
Bayesian Ridge	0.54	0.74	0.38	0.32	0.31 ± 0.06
Lasso	0.56	0.78	0.31	0.30	0.28 ± 0.04

**Table 2:** Results for the consensus model, reporting the MAE, RMSE, and R^2^ for the external test.

Integrated	MAE	RMSE	R2
Extra Trees GB XGB	0.31	0.45	0.78

**Table 3:** An applicability domain analysis for the Extra Trees, Gradient Boosting, and XGB models using centroid-based and bounding box methods.

	Bounding Box	Centroid-Based
Extra Trees	0.77	0.78
GB	0.76	0.77
XGB	0.75	0.76
Integrated	0.77	0.78

## Data Availability

The data and the developed models are freely accessible at https://github.com/EdoardoVigano/AntioxidantActivity. (accessed on 1 May 2025).

## Abbreviations

The following abbreviations are used in this manuscript:

Adaboost	Adaptive Boosting Digital
AD	Applicability domain
DPPH	1,1-Diphenyl-2-picrylhydrazyl
GB	Gradient Boosting
IC50	Half-maximal inhibitory concentration
k-NN	k-nearest neighbor
MAE	Mean Absolute Error
MI	Mutual information
NAMs	New approach methodologies
PBPK	Physiologically Based Pharmacokinetic
QSAR	Quantitative Structure–Activity Relationship
R&D	Research and Development
RF	Random Forest
ROS	Reactive Oxygen Species
RMSE	Root-Mean-Squared Error
XGB	eXtreme Gradient Boosting
